# Parallax Inference for Robust Temporal Monocular Depth Estimation in Unstructured Environments

**DOI:** 10.3390/s22239374

**Published:** 2022-12-01

**Authors:** Michaël Fonder, Damien Ernst, Marc Van Droogenbroeck

**Affiliations:** 1 Department of Electrical Engineering and Computer Science, University of Liège, 4000 Liège, Belgium; 2 Laboratoire Traitement et Communication de l’Information (LTCI), Télécom Paris, 91120 Palaiseau, France; 3 Institut Polytechnique de Paris, 91120 Palaiseau, France

**Keywords:** depth estimation, deep learning, unmanned vehicles, parallax

## Abstract

Estimating the distance to objects is crucial for autonomous vehicles, but cost, weight or power constraints sometimes prevent the use of dedicated depth sensors. In this case, the distance has to be estimated from on-board mounted RGB cameras, which is a complex task especially for environments such as natural outdoor landscapes. In this paper, we present a new depth estimation method suitable for use in such landscapes. First, we establish a bijective relationship between depth and the visual parallax of two consecutive frames and show how to exploit it to perform motion-invariant pixel-wise depth estimation. Then, we detail our architecture which is based on a pyramidal convolutional neural network where each level refines an input parallax map estimate by using two customized cost volumes. We use these cost volumes to leverage the visual spatio-temporal constraints imposed by motion and make the network robust for varied scenes. We benchmarked our approach both in test and generalization modes on public datasets featuring synthetic camera trajectories recorded in a wide variety of outdoor scenes. Results show that our network outperforms the state of the art on these datasets, while also performing well on a standard depth estimation benchmark.

## 1. Introduction

Estimating accurate dense depth maps is an essential task for unmanned vehicles [1,2]. Having access to the distance that separates them from objects of the environment is indeed a prerequisite for most obstacle avoidance and trajectory planning algorithms [3,4,5]. To the best of our knowledge, the only reliable long-range distance sensors suitable for outdoors are heavy, bulky, power consuming, and expensive, which makes them unsuitable for use on vehicles such as drones where weight, size, and power availability are constrained. Therefore, these vehicles need to infer distances by other means. One way to achieve this is to replace the distance sensor with an algorithm that estimates depth from RGB pictures captured by an on-board-mounted camera, as done by Kim et al. [4] for instance.

Small and lightweight unmanned ground or aerial vehicles are perfect devices to reach places otherwise barely accessible and, hence, are often used to venture off road in environments where direct cues about distance, such as object structures, are unknown. Natural landscapes are examples of such environments whose elements (vegetation, ground and relief) do not exhibit normalized structures or patterns. However, datasets suitable to study the task of depth estimation in environments with little structure—we call them “unstructured environments” in the following—were proposed only recently (see Fonder and Van Droogenbroeck [6], Wang et al. [7]). As a result, existing methods for outdoor depth estimation [8,9,10,11,12] are benchmarked against older datasets that only target autonomous driving applications in urban environments.

These methods are all trained to directly infer depth or its inverse, known as disparity. As the motion of a car on a road is strongly constrained and urban areas contain many objects with a specific structure (cars, roads, signs, buildings, etc), the autonomous driving datasets mainly feature constrained trajectories and environments. Since the structure and semantics of elements in a scene likely provide direct cues about depth, it is uncertain if existing methods perform well in environments where such cues are not available. Furthermore, learning to directly infer depth or disparity reduces the capability of networks to generalize in environments where the depth distribution differs from the one used for training.

In this paper, we address the challenging task of estimating depth in unstructured environments. To overcome the shortcomings of existing methods, we introduce a notion of visual parallax, the frame-to-frame displacement of a pixel in an image sequence. With our formulation, the visual parallax is decoupled from the depth distribution thanks to the camera motion. This allows us to build a deep neural network based on plane-sweeping cost volumes that performs well when inferring depth both in specific environments and in generalization.

As highlighted by Schröppel et al. [13], building plane-sweeping cost volumes from depth or disparity intervals requires two arbitrary parameters that constraint the performance and the generalization properties of the underlying network. In this work, we overcome the need to determine arbitrary parameters by building plane-sweeping cost volumes from parallax intervals.

The related work and the precise formulation of the task we address in this paper are presented in Section 2 and Section 3, respectively. Then, in Section 4, we describe our dedicated depth estimation method. In Section 5, we detail our experimental setup which is aimed at evaluating methods on unstructured environments and in generalization. We also present our results and discuss our method in this section. Section 6 concludes the paper.

Our main contributions can be summarized as follows:

1. We define a notion of visual parallax between two frames from a generic six-degree-of-freedom (6-DoF) camera motion, and present a way to build cost volumes with this parallax;
2. We present a novel lightweight multi-level architecture, called M4Depth, that is based on these cost volumes, designed to perform end-to-end depth estimation on video streams acquired in unstructured environments, and suitable for real-time applications;
3. It is shown that M4Depth, is state-of-the-art on the Mid-Air dataset [6], that it has good performances on the KITTI dataset [14], and that it outperforms existing methods in a generalization setup on the TartanAir dataset [7].

## 2. Related Work

Related works are presented according to four different categories.

**(1) Depth from a single image.** Estimating depth from a single RGB image is an old and well-established principle. If the first methods were fully handcrafted [15], the growth of machine learning and the development of CNNs has impacted on the field of depth estimation through the introduction of new methods such as Monodepth [16], Monodepth2 [17], or the method proposed by Poggi et al. [18] that have led to massive improvements in the quality of the depth estimates. Methods based on vision transformer networks, such as DPT [19] or AdaBins [20], push the performance even further and are currently the state-of-the-art in the field.

**Figure 1:**
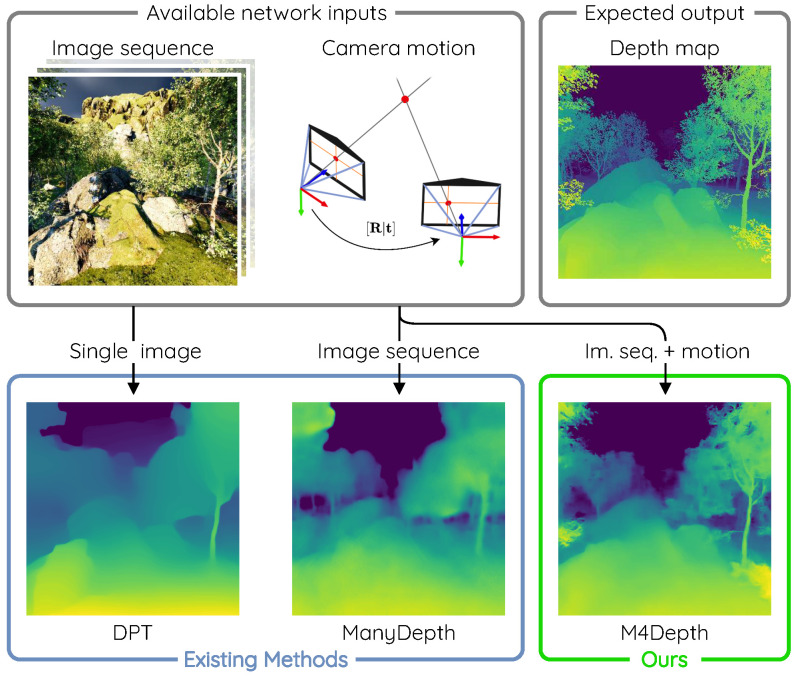
State-of-the-art depth estimation methods such as DPT [19,21] or ManyDepth [22] struggle to produce accurate estimates in cluttered and natural environments. Our method, called M4Depth, outperforms existing methods in these instances and generalizes well to unknown environments.

Recent surveys [9,10,11,12] present summarized descriptions and comparisons of single image depth estimation methods. The main observation made in these surveys is that estimating depth from a single image remains difficult, especially for autonomous vehicle applications. Since the problem is ill-posed, networks have to heavily rely on priors to compute a suitable proposal. Such dependency on priors leads to a lack of robustness and generalization. Therefore, methods of this family need to be fine-tuned for every new scenario or environment encountered in order to produce good estimates. Despite their massive parameter count, transformers are no exception to these observations, as illustrated in Figure 1.

**(2) Depth from an image sequence.** Methods exist that include recurrence in networks to make use of temporal information for improving estimates [23,24,25,26,27]. They are mainly adaptations of existing architectures through the addition or modification of specific layers. As such, these methods do not make direct use of motion-induced constraints.

In their method named ManyDepth, Watson et al. [22] avoided this issue by relying, among other things, on a model for motion and a cost volume built with the plane-sweeping method [28,29]. Xing et al. [30] avoided the need for explicit motion modeling by using the planar parallax geometry [31,32]. The latest method however consists in a complex pipeline that explicitly relies on structure in the environment, which makes it unusable for our problem.

By design, methods independent of the camera motion are unable to estimate the proper scale for depth without relying on any prior knowledge about the structure of the scene. In addition, the scale estimated for the outputs is likely to drift over the sequence because there is no way to validate the proper scale at any time. This is problematic for autonomous vehicles moving in unstructured environments.

**(3) Use of motion information.** When motion is used by depth estimation methods, it is mostly exploited to build a loss function for self-supervised training [17,24,25,33,34]. In these cases, motion and depth are learnt by two independent networks in an unsupervised fashion and depth is still estimated without any clue about motion. As the core of these depth estimation networks does not change compared to methods simply working on sequences, their estimations suffer from the same issues as the ones produced by methods that do not use motion.

One notable exception is the idea proposed by Luo et al. [35]. Their method uses a self-supervised loss based on motion estimation to fine-tune the network at test time. It achieves outstanding performance, but at the cost of a large computational burden. Furthermore, this method cannot estimate depth before the whole image sequence is available, meaning that it only operates in an offline mode and makes it inappropriate for autonomous vehicle applications.

**(4) 3-D reconstruction.** Structure from motion (SfM) and multi-view stereo (MVS) are two research fields that have developed in parallel with depth estimation. The idea is to reconstruct 3-D shapes from a set of RGB images that capture the scene from different points of view under specific hypothesis (MVS requires known camera poses, SfM does not). Reconstruction is achieved by explicitly expressing the relative camera position between the images of the set. Approaches for performing this task are varied [36,37] and are, by their nature, often unsuitable for real-time depth estimation. However, some are adaptable for depth estimation on sequences [38,39,40], while others are specifically designed to work on image sequences in real time [41,42].

The approach proposed by Düzçeker et al. [41] and the method called DeepV2D by Teed and Deng [42] are similar. They both propose a three-stage network. Their stages are an image-encoding network followed by the computation of a cost volume that is finally processed by a depth estimation network. The purpose of the cost volume consists of providing the costs for matching a point in an image with a series of candidates in another image. The cost volume of both methods is built by a plane-sweeping method [28,29].

As highlighted by Schröppel et al. [13], the multi-frame methods that rely on plane-sweeping cost volumes share a common shortcoming. They build their cost volumes from depth or disparity intervals, which requires two arbitrary parameters: (1) the maximum range, and (2) the quantization step along this range. The maximum range has to be known at the time of training or fine-tuning, which prevents dynamic adaptations to new depth distributions. The quantization step, that is the range divided by the number of samples along the range, is an important parameter for determining the performance of the network. A large quantization step degrades the depth resolution, hence the performance of a network, while taking a smaller quantization step will increase the inference time without any guarantee of improving the final result.

## 3. Problem Statement

We now present the technicalities of the problem we want to solve. We consider a camera rigidly attached to a vehicle moving within an unknown static environment. The intrinsic parameters of the camera are supposed to be known and constant. We introduce the following components and notations:

- $I_{t}$ is an RGB image of size H×W recorded by the camera at time step *t*. Images have the following properties: (1) motion blur and rolling shutter artifacts are negligible; (2) the camera focal length *f* is known and constant during a flight; (3) the camera shutter speed and gain are unknown, and can change over time;
- $T_{t}$ is the transformation matrix encoding the motion of the optical center of the camera from time step t−1 to *t*. As this matrix is computed for monitoring the state of the vehicle, we assume that it is available for our method as well.
- $z_{ij,t}$ is the *z* coordinate (in meters) of the point recorded by the pixel at coordinates (i,j) of the frame $I_{t}$ with respect to the camera coordinate system.

Using these notations, a depth map $d_{t}$ is an array of $z_{ij,t}$ values with ij∈{1,…,W}×{1,…,H}.

We denote by $h_{t}$ the complete series of image frames and camera motions up to time step *t*. We define a set D of functions *D* that are able to estimate a depth map d from $h_{t}$, that is $\hat{d_{t}}=D\left(h_{t}\right)$, such that $\;D\in D,\;\mathrm{with}\;h_{t}=\left[I_{0},\left[I_{1},T_{1}\right],\;\ldots \;,\;\left[I_{t},T_{t}\right]\right]$. Our objective is to a find function *D* in this set that best estimates $d_{t}$.

Since collision avoidance is essential for autonomous vehicle applications, errors in the estimate of distance for closer objects should have a higher impact than errors occurring for objects in the background of the scene. This is taken care of by constructing a dedicated loss function for training and by minimizing the error relative to the distance of the object. During testing, we will use the set of performance metrics defined by Eigen et al. [43] to better grasp the behavior of our method.

**Our baseline.** Based on the related work, we have selected a representative set of existing methods for which the training code is available, as given in Table 1; they constitute the baseline for our performance tests. In this table, we indicate for each method, respectively, the nature of its supervision mode, if it is based on a single or multiple frames, if it is recurrent, how it deals with the camera pose, and if weights for the KITTI are provided by the authors.

## 4. Description of a New Method for Depth Estimation

Like other previous works, our method, named *Motion for Depth* (aka M4Depth), is based on a multi-level architecture that relies on cost volumes and that is trainable in an end-to-end fashion. The key novelty is that the network is designed to infer a parallax map, which is converted into a depth map by using motion information. The parallax map has several interesting properties that should make M4Depth more robust in generalization. This is described in the next section.

### 4.1. Deriving the Bijective Relation between Depth and Visual Parallax

The apparent displacement of static objects between images captured with a moving camera is the parallax effect. Parallax is defined for a generic frame-to-frame transformation, and degenerates into standard stereo disparity when the frame-to-frame transformation amounts to a translation along the camera *x* or *y* axis. Like disparity, parallax conveys information about the distance of objects from the camera, but for an unconstrained camera baseline.

Previous works using parallax geometry [30,31,32] assume that frame-to-frame point correspondence, hence parallax, is known to derive 3-D rigidity constraint between pairs of points for recovering the 3-D scene structure without using the camera motion. Our method does the opposite; it uses the constraints imposed by motion, whose parameters are provided by the on-board inertial measurement unit, and geometry to guide the inference of parallax along the epipolar lines. In the following, we establish that depth is a linear function of the inverse of the parallax when the latter is defined in a specific way.

Our notion of parallax, new to the best of our knowledge, denoted by ρ, is established as follows. The transformation matrix $T_{t}$ formalizing the known physical camera motion with 6 DoF between consecutive frames of the video stream can be broken down into a rotation matrix $R_{t}$ and a 3-D translation vector $t_{t}={\left[\begin{array}{ccc} t_{x} & t_{y} & t_{z} \end{array}\right]}^{T}$. Using the classical pinhole camera model, a point *P* in space seen by the camera at two different time instants *t* and t−1, and projected at coordinates $\left(i_{t},j_{t}\right)$ in the current frame *t* is linked to its previous coordinates $\left(i_{t-1},j_{t-1}\right)$ in frame at time t−1 by the motion $T_{t}$ as follows:

(1) $$z_{i_{t-1}j_{t-1}}\left[\begin{array}{c} i_{t-1}\\ j_{t-1}\\ 1 \end{array}\right]=K\left(R_{t}z_{i_{t}j_{t}}\left[\begin{array}{c} i_{t}/f_{x}\\ j_{t}/f_{y}\\ 1 \end{array}\right]+t_{t}\right)\;,$$

where $z_{i_{t}j_{t}}=d_{t}\left(i_{t},j_{t}\right)$ is the depth of the point *P* at time *t*, and K is a camera calibration matrix, which is identical at times *t* and t−1. It is possible to simplify the expression of the 3×3 K matrix to:

(2) $$K=\mathrm{diag}(f_{x},f_{y},1)\;,$$

with $f_{x}$ and $f_{y}$ being the focal lengths along the *x* and *y* axes respectively. This assumes that the coordinates (i,j) are expressed directly with respect to the principal point of the sensor $\left(c_{x},c_{y}\right)$ and that the skew parameter is negligible.

Before defining our visual parallax, we rewrite Equation (1) as

(3) $$z_{i_{t-1}j_{t-1}}\left[\begin{array}{c} i_{t-1}\\ j_{t-1}\\ 1 \end{array}\right]=z_{V}\;z_{i_{t}j_{t}}\left[\begin{array}{c} i_{V}\\ j_{V}\\ 1 \end{array}\right]+\left[\begin{array}{c} f_{x}t_{x}\\ f_{y}t_{y}\\ t_{z} \end{array}\right]\;,$$

with

(4) $${\left[z_{V}i_{V},\;z_{V}j_{V},\;z_{V}\right]}^{T}=\;K\;R\;{\left[i_{t}/f_{x},\;j_{t}/f_{y},\;1\right]}^{T}\;.$$

From this equation, we can see that $\left(i_{V},j_{V}\right)$ are the coordinates of the point *P* in the plane of a virtual camera *V* whose origin is the same as the camera at time *t* but with the orientation of the camera at time t−1.

We now introduce the parallax map $\rho_{t}$, where the pixelwise parallax $\rho_{i_{t}j_{t}}=\rho_{t}\left(i_{t},j_{t}\right)$ is defined as the Euclidean norm,

(5) $$\rho_{i_{t}j_{t}}=\sqrt{\Delta_{i_{t}}^{2}+\Delta_{j_{t}}^{2}},$$

where

(6) $$\left[\begin{array}{c} \Delta_{i_{t}}\\ \Delta_{j_{t}} \end{array}\right]=\left[\begin{array}{c} i_{t-1}-i_{V}\\ j_{t-1}-j_{V} \end{array}\right]\;.$$

With this definition, the parallax is only a function of perceived pixel motion. It is therefore invariant to the particular combination of depth and camera motion.

After reorganization, using Equation (3) and simplification, we get:

(7) $$\left[\begin{array}{c} \Delta_{i_{t}}\\ \Delta_{j_{t}} \end{array}\right]=\frac{1}{z_{i_{t}j_{t}}\;z_{V}+t_{z}}\left[\begin{array}{c} f_{x}t_{x}-t_{z}i_{V}\\ f_{y}t_{y}-t_{z}j_{V} \end{array}\right]\;.$$

By taking into account the physics of a scene and the camera motion of an autonomous vehicle, it can be shown that $z_{i_{t}j_{t}}\;z_{V}+t_{z}$ should rarely be negative. As a result, the parallax $\rho_{i_{t}j_{t}}$ can be computed as follows:

(8) $$\begin{array}{cc} \rho_{i_{t}j_{t}}= & \frac{\sqrt{{\left(f_{x}t_{x}-t_{z}i_{V}\right)}^{2}+{\left(f_{y}t_{y}-t_{z}j_{V}\right)}^{2}}}{z_{i_{t}j_{t}}\;z_{V}+t_{z}}\;. \end{array}$$

This bijective expression links the parallax for a pixel to the depth of the corresponding point in space. Since parallax can be estimated from the RGB content of two consecutive images, we have a means to estimate the depth by inverting the equation, yielding:

(9) $$z_{i_{t}j_{t}}=\frac{\sqrt{{\left(f_{x}t_{x}-t_{z}i_{V}\right)}^{2}+{\left(f_{y}t_{y}-t_{z}j_{V}\right)}^{2}}}{\rho_{i_{t}j_{t}}\;z_{V}}-\frac{t_{z}}{z_{V}}\;.$$

As expected, this expression becomes identical to the definition of the standard stereo disparity when the camera only moves along the *x* or *y* axis.

In practice, there are different ways to estimate $\rho_{i_{t}j_{t}}$, and in our method, we build various proposals for $\rho_{i_{t}j_{t}}$ and let the network use them to compute the best estimate. Note that, once a parallax map $\rho_{t}$ has been estimated, the $\left(i_{t-1},j_{t-1}\right)$ coordinates are given by a function Ψ, parametrized as follows

(10) $$\left(i_{t-1},j_{t-1}\right)=\Psi \left(i_{t},j_{t},T_{t},\rho_{t}\right)\;.$$

These $\left(i_{t-1},j_{t-1}\right)$ coordinates are defined on a continuous space instead of a discrete grid.

### 4.2. Definition of the Network

Our primary motivation for inferring parallax rather than depth directly is driven by the need to produce a method that is robust, even in unseen environments. Training a network to infer depths drawn from a given data distribution will tie it to this distribution. Our formulation for the parallax consists of decoupling the value to infer from depth thanks to motion, and allows one to map many depth values to a same parallax value. As a result, a single learnt parallax distribution can represent many depth distributions, which is a desirable ability for robustness and generalization.

As for optical flow, estimating the parallax can be performed iteratively. Instead of simply iterating on a full network as proposed in [39], we approach the iterative process as a multi-scale pyramidal network, as PWC-Net [44]. By doing so, we embed the iterative process in the architecture itself. This architecture is an adaptation of the U-Net encoder-decoder with skip connections [45], where each level *l* of the decoder has to produce an estimate for the desired output, which in our case is a parallax map. In the decoder, the estimate produced at one level is forwarded to feed the next level to be refined. The levels of this type of architecture are generic and can be stacked at will to obtain the desired network depth.

**Figure 2:**
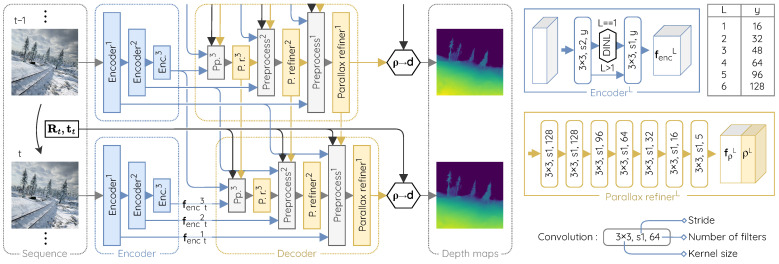
Architecture overview of M4Depth (with three levels here), fed by two consecutive frames and the camera motion. Each parallax refiner produces a parallax estimate and learnable parallax features. All convolutions are followed by a leaky ReLU activation unit [47], except for the ones producing a parallax estimate. To ease the convergence, parallax values are encoded in the log-space. Details of the preprocessing unit are given in Figure 3.

Our architecture, illustrated in Figure 2, uses the same standard encoder network as PWC-Net [44] with the only exception that we add a Domain-Invariant Normalization layer (DINL) [46] after the first convolutional layer. We use it to increase the robustness of the network to varied colors, contrasts and luminosity conditions without increasing the number of convolutional filters.

At each level *L* of the decoder, a small convolutional subnetwork is in charge of refining the parallax map. We named it the parallax refiner. Its inputs are the upscaled parallax estimate made by the previous decoder level in the architecture and a series of preprocessed data generated by a preprocessing unit.

**Figure 3:**
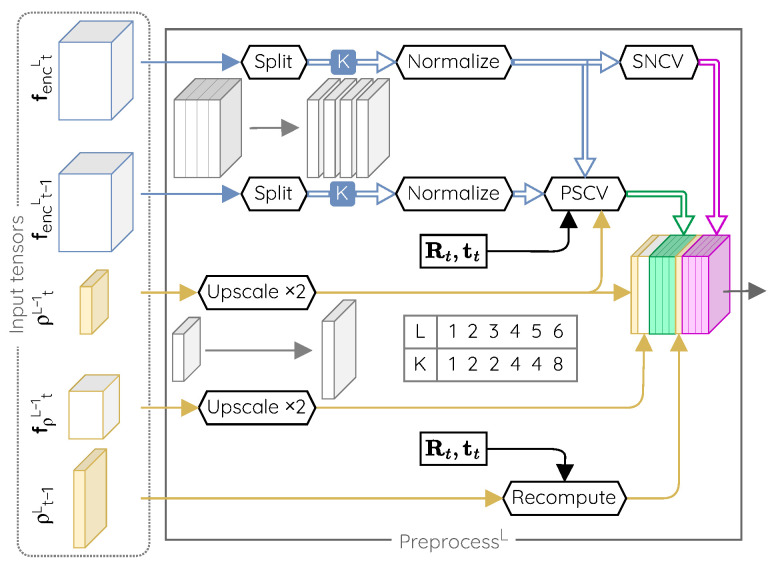
Details of the operations performed by our preprocessing units. Its building blocks do not feature any learnable parameters; they are detailed in Section 4.2. The split layer subdivides feature vectors in K sub-vectors to be processed in parallel in subsequent steps. We give the value of K that we use for an architecture with six levels.

The preprocessing unit is illustrated in Figure 3. It is made of fixed operations and has no learnable parameters. Its purpose is to prepare the input for the next parallax refiner.

In short, the preprocessor has two main purposes. First, it adapts the vectors of the feature maps produced by the encoders to make the network robust to unknown visual inputs. For that, it uses these data alongside camera motion to build two distinct cost volumes, the Parallax Sweeping Cost Volume (PSCV) and the Spatial Neighborhood Cost Volume (SNCV). Second, it recomputes the parallax estimate obtained for the previous time by adjusting it to the camera motion. These data are then concatenated and forwarded to the parallax refiner.

#### Description of the Building Blocks of the Preprocessing Unit

In the following, we describe the components of the preprocessing unit and motivate their use.

**Split and normalize layers.** The use of leaky ReLU activation units in the encoder can lead to feature maps containing plenty of small values. While classification or segmentation networks rely on the raw value of each entry in a feature vector, our network relies on the relative differences between neighboring feature vectors through the use of cost volumes. To achieve good generalization properties, this relative difference should remain significant in all situations. The split and normalize layers ensure that this is the case.

The split layer subdivides feature vectors in K sub-vectors to be processed in parallel in subsequent layers. It provides the network with the ability to decouple the relative importance of specific features within a same vector by assigning them to different sub-vectors.

The normalize layer normalizes the features of a same sub-vector and therefore levels the difference in magnitude of different sub-vectors. This is beneficial for the parallax refiner layers as this normalization leads the outputs of the cost volumes to span to a known pre-defined range. It also allows a full use of the information embedded in sub-vectors whose magnitude is very small because of the leaky ReLU activation units.

**Recompute layer.** The parallax values estimated by the network are specific to the motion occurring between two given frames. By using the set of equations developed in Section 4.1 and if the camera motion is known, it is possible to compute the parallax values that should be observed at a given time step from a previous parallax estimate. The purpose of the recompute layer is to update the parallax values estimated for the previous frame to provide a hint in the form of a first estimate of the parallax values for the current frame.

**Spatial Neighborhood Cost Volume (SNCV).** This cost volume is computed from a single feature map f and is a form of spatial autocorrelation. Each pixel of the cost volume is assigned the costs of matching the feature vector located at the same location in the feature map with the neighboring feature vectors located within a given range *r* of the considered location,

(11) $${\mathrm{SNCV}}_{r}\left(f\right)\left(i,j\right)=\left[\;\cos t\left(f(i,j),f(i+p,j+q)\right)\;\forall \;p,q\in \left\{-r,\ldots ,r\right\}\;\right]\;,$$

where the cost of matching two vectors $x_{1}$ and $x_{2}$ of dimension *N* is defined as their correlation [48,49].

(12) $$\cos t\left(x_{1},x_{2}\right)=\frac{1}{N}x_{1}^{T}x_{2}\;.$$

The SNCV gives an indication about the two-dimensional spatial structure of the scene captured by the features of the encoder. By design, it is impossible to recover the feature vectors that led to a given cost value. Network parameters trained with this cost metric will therefore be invariant to changes in the input feature vectors if they lead to the same cost value. This can help us to obtain a robust and generalizable depth estimation network, which was not achievable by forwarding the feature map directly.

**Parallax Sweeping Cost Volume (PSCV).** This cost volume is computed from two consecutive feature maps $f_{t-1}$ and $f_{t}$, and a parallax map estimate ${\hat{\rho }}_{t}$ (see left of Figure 3). For each pixel, the cost volume assigns the cost of matching the feature vector located at the same place in $f_{t}$ with the corresponding reprojected feature vectors from $f_{t-1}$ according to:

(13) $${\mathrm{PSCV}}_{\delta }\left(f_{t},f_{t-1},{\hat{\rho }}_{t}\right)\left(i,j\right)=\left[\cos t\left(f_{t}\left(i,j\right),f_{\mathrm{reproj}}\left(i,j,\Delta_{\rho }\right)\right)\;\forall \;\Delta_{\rho }\in \left\{-\delta ,\ldots ,\delta \right\}\right]\;.$$

In that expression, the feature map $f_{t-1}$ is reprojected for a range δ of parallax values equally distributed around a given estimate, that is

(14) $$f_{\mathrm{reproj}}\left(i,j,\Delta_{\rho }\right)=f_{t-1}\left(\Psi \left(i,j,T_{t},\max\left(\epsilon ,{\hat{\rho }}_{t}+\Delta_{\rho }\right)\right)\right)\;,$$

where Ψ is given by Equation (10), and ϵ>0. In this expression, $f_{\mathrm{reproj}}$ is interpolated from $f_{t-1}$ since Ψ returns real coordinates, and $\max\left(\epsilon ,{\hat{\rho }}_{t}+\Delta_{\rho }\right)$ ensures the positiveness of the parallax used for computing the reprojection. Each vector element of the cost volume corresponds to one given parallax correction with respect to the provided estimate. Browsing through a range of parallax values for each pixel creates a series of candidates for the corresponding reprojected point. By searching for the reprojected candidate that is the most similar to the visual information observed at time step *t*, it is possible to assess which parallax is the most likely to be associated to each pixel.

Building the cost volumes from parallax intervals eliminates the contextual and arbitrary choices required otherwise (i.e., the range and the quantization step). As the parallax is defined within the image space, it is indeed bound to the image resolution. A good step size is therefore always 1 (pixel) and the maximum parallax value that can be encountered is equal to the diagonal of the image (in pixels). Since we are using a pyramidal architecture, a range δ at the *L*-th level is equivalent to a range $\delta 2^{L}$ in the original image. By stacking *N* levels, our architecture can theoretically cover a range of $\delta (2^{N+1}-2)$ pixels while needing a total of only N(2δ+1) samples to obtain a pixelwise resolution.

### 4.3. Loss Function Definition

Since the levels of our architecture are stackable at will, the architecture can have any depth. We now detail our loss function for a network that is made of *M* levels.

As in previous works [50,51,52], we use a multi-scale loss function. For each frame and each level, we compute the $L_{1}$ distance on the logarithm of the depths resulting from the conversion of parallax estimates using Equation (9). The logarithm leads to a scale invariant loss function [43] and the use of an $L_{1}$ distance is motivated by its good convergence properties [53]. Since intermediate depth maps have a lower resolution, ground truths are resized by bilinear interpolation to match the dimensions of the estimates. The resulting terms are aggregated through a weighted sum, yielding

(15) $$L_{t}=\frac{1}{HW}\sum_{l=1}^{M}\sum_{z_{ij}\in d_{t}^{l}}2^{l+1}\;\left|\log\left(z_{ij}\right)-\log\left({\hat{z}}_{ij}\right)\right|\;.$$

The total loss for the sequence is defined as the average of the loss computed for each of its time steps.

## 5. Experiments

In this section, we present three experiments to analyze the performance of our method. For each of them, we detail the chosen dataset, the training (if appropriate), and discuss the results. Our first and second series of experiments aim to assess the performance in unstructured environments, as driven by our problem statement, and on a standard benchmark, respectively. The experiments present comparisons with baseline methods that might significantly differ, in terms of training procedure, number of parameters, etc. In order to disentangle the intrinsics of the training phase, we have devoted our third series of experiments to generalization tests.

We use the metrics from Eigen et al. [43] for depth maps capped at 80 m to compare the performance of each method. Additionally, we replicate the experiments performed on M4Depth with PWC-Net [44] to evaluate the benefits of our proposal over its parent. As PWC-Net is an optical flow network, we use Equation (1) to obtain the frame-to-frame optical flow from depth and motion for training the network. During testing, we compute depth by assuming that the length of the optical flow vectors corresponds to the visual parallax.

Please note that we provide all the codes of M4Depth and tested methods on GitHub (https://github.com/michael-fonder/M4Depth), both for training and testing modes.

### 5.1. Unstructured Environments

For our first experiment, we compare the performance of our method with those of the state-of-the-art on a dataset featuring unstructured environments.

**Mid-Air dataset.** For this experiment, we used Mid-Air [6]. This synthetic dataset consists of a set of drone trajectories recorded in large, unstructured, and static environments under varied weather and lighting conditions. All trajectories were recorded in different places of the environments, which means that there was little overlap between the visual content of two individual trajectories. This allows one to build a test set whose content is not present in the training set while belonging to the same data distribution. In addition, Mid-Air meets all the assumptions of our problem statement (see Section 3), which makes it a perfect choice.

The first performance reported on Mid-Air for depth estimation was provided recently by Miclea and Nedevschi [54]. The authors of this paper do not, however, provide the details required to reproduce their train and test splits and their results. As a result, we have to define our own splits.

The dataset features 192 trajectories. We selected one in three to create our test set, which was more varied than the small test set suggested in the original paper [6]. The frame rate was subsampled by a factor of four (from 25 to 6.25 fps) to increase the apparent motion between two frames. For all our experiments, images and depth maps were resized to a size of 384×384 pixels. We used bilinear interpolation to resize color images and the nearest-neighbor method for depth maps.

**Training.** We used the He initialization [55] for our variables and the Adam optimizer [56] for the training itself. We used the default moment parameters for the latter ($\beta_{1}=0.9$, $\beta_{2}=0.999$). The learning rate was set to $10^{-4}$. We trained our network with six levels. All our trainings were performed on sequences of four time steps and with a batch size of three sequences. The network was trained on a GPU with 16GB of VRAM for 220k iterations. After each epoch, we computed the performance of the network on the validation set of the KITTI dataset to avoid any overfitting, and keep a copy of the best set of weights to be used for the tests after the training.

A series of data augmentation steps were performed on each sequence during the training to boost the robustness of our trained network to visual novelties. More precisely, we applied the same random brightness, contrast, hue, and saturation change to all the RGB images of a sequence and the colors of a sequence were inverted with a 50% probability. Finally, we randomly rotated the data of the sequence by a multiple of 90 degrees around the *z*-axis of the camera when training on Mid-Air. With these settings, a training takes approximately 30 h.

Because of the lack of reproducible performances reported on Mid-Air, we had to train a selection of state-of-the-art methods drawn in Table 1 to build a baseline. The training details for the chosen methods are given in the Appendix A. We could not guarantee obtaining the best performance out of DeepV2D [42] because of the importance of its hyper-parameters and the excessive duration of its training time. We, therefore, decided to discard it for this experiment.

**Results.** The results are reported in Table 2. In this table and following tables, the best score for a metric is highlighted in bold and the second best is underlined.

**Figure 4:**
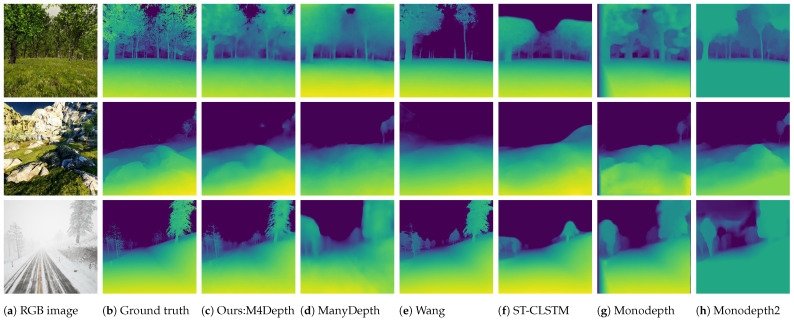
Comparison of the depth maps estimated by M4Depth and baseline methods. M4Depth recovers depth details more accurately than baseline methods.

Globally, it appears that M4Depth outperforms the baseline methods. However, it slightly underperforms on the relative performance metrics when compared to PWC-Net. This observation, compared with the excellent performances on other metrics, indicates that our network tends to overestimate depth more often than other methods. A qualitative comparison of the outputs of the different methods is shown in Figure 4. From this figure, we observe that although M4Depth lacks details in areas with sharp depth transitions, it recovers depth details more accurately than baseline methods, even for challenging scene elements such as forests or unstructured terrain.

### 5.2. Standard Depth Estimation Benchmark

The purpose of the second experiment was to assess the performance on a standard depth estimation benchmark.

**KITTI dataset [14].** Most real datasets that provide RGB+D and motion data focus on cars driving in partially dynamic urban environments [14,57,58]. In this field, KITTI is the reference benchmark dataset when evaluating the performance of a depth estimation method. KITTI is not fully compliant with our problem statement: it has incomplete depth maps, there are some moving objects, and the camera has only three degrees of freedom, etc. Despite that, it is a good practical choice for performing tests on real data.

We used the dataset split proposed by Eigen et al. [43]. The camera pose was estimated by a combined GPS-inertial unit and was therefore subject to measurement imperfections. Since a few samples were recorded in urban canyons where poor GPS reception induced erratic pose estimates, and as our method requires reliable pose estimates, we discarded these problematic samples from the splits. Additionally, we also subsampled the frame rate by a factor of two (from 10 to 5 fps) to roughly match the one of our Mid-Air sets. Finally, images were resized to 256×768 pixels.

**Training.** For tests on KITTI, we reused the weights of the network with six levels trained on Mid-Air and fine-tuned them for 20k additional iterations on a 50–50% mix of KITTI and Mid-Air samples. The fine-tuning was required to train our network to deal with large areas with poor textures and frame-to-frame illumination changes as these characteristics are not present in Mid-Air. As the ground-truth depth maps for KITTI were generated from Lidar measurements, they were sparse and fine details were missing in the ground truths. Shortcomings created by these imperfections can be mitigated by fine-tuning on both datasets. During the fine-tuning, we also performed random color augmentation on the sequences. With these settings, the fine-tuning takes three hours.

**Results.** The performance of M4Depth with six levels on the KITTI dataset is reported in Table 3.

We observe that M4Depth has similar performances to current state-of-the-art methods. As expected, instances with dynamic elements or poor GPS localization lead to degraded performances. These results, however, prove that M4Depth also works with real data despite their imperfections.

**Figure 5:**
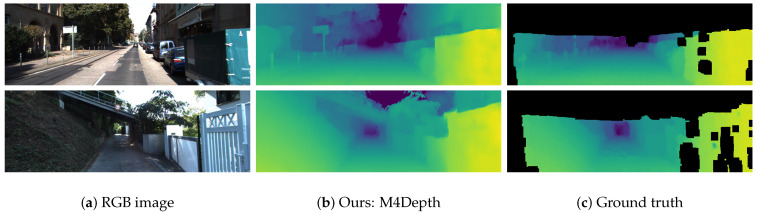
Comparison of depth maps estimated by M4Depth on the KITTI dataset with the corresponding interpolated ground truth.

An overview of the outputs of our method on KITTI is shown in Figure 5. M4Depth appears to preserve fine details, and to reliably estimate depth even in areas with less texture or for shiny objects such as cars.

### 5.3. Generalization

In this last experiment, we wanted to evaluate the generalization capabilities of all the methods. For this, we wanted to use static scenes that were semantically close to either the Mid-Air dataset (natural unstructured environments) or the KITTI dataset (urban environments), and test the performance of the method trained on Mid-Air (respectively KITTI) on the selected unstructured (respectively urban) scenes without any fine-tuning.

As we wanted to focus only on the generalization performance for depth estimation, we bypassed the pose estimation network for ManyDepth and DeepV2D, and used the ground-truth motion to generate the depth maps with these methods. Additionally, the depth maps produced by baseline methods were not guaranteed to be at the correct scale. To alleviate this issue in performance tests, we applied a median scaling to the depth maps of baseline methods.

**TartanAir dataset [7].** For this experiment, we used TartanAir. It is a synthetic dataset consisting of trajectories recorded by a free-flying camera in a series of different environment scenes. With each scene being relatively small in size, there is a lot of overlap in the visual content recorded for different trajectories within a same scene. As such, assembling clearly separated train and test sets drawn from the same data distribution is not possible. Despite this drawback, the diversity of the scenes makes TartanAir an interesting choice for testing the generalization capabilities of methods.

For the generalization test from the Mid-Air dataset, we selected the “Gascola” and “season forest winter” scenes of TartanAir and used the weights trained for the baseline. For the one from the KITTI dataset, we selected the “Neighborhood” and “Old Town” scenes and used the pre-trained weights released by the authors of the methods.

We resized the images of this dataset to 384×576 pixels and subsampled the frame rate by a factor of two. Additionally, some scenes appeared to have large, underexposed areas where there was no color information in the RGB frames. Having large pitch-black areas in an RGB image is unrealistic in practice as cameras dynamically adapt their shutter speed depending on the exposure of the scene. To prevent the errors made by depth estimation methods in these areas from dominating the performance analysis, we discarded all the pixels for which the color in the RGB image had a value equal to zero.

**Results.** The results of our experiments are reported in Table 4 and Table 5. Overall M4Depth outperforms the other methods with a significant margin both for structured and unstructured environments. As on Mid-Air, PWC-Net slightly outperforms M4Depth on some relative metrics, but not for both sequences. It is worth noting that the hierarchy of the performances has completely changed between the test on KITTI and the one in generalization as our method outperforms DeepV2D [42] on the latter. These results therefore show the better generalization capability of M4Depth when compared to state-of-the-art methods.

**Figure 6:**
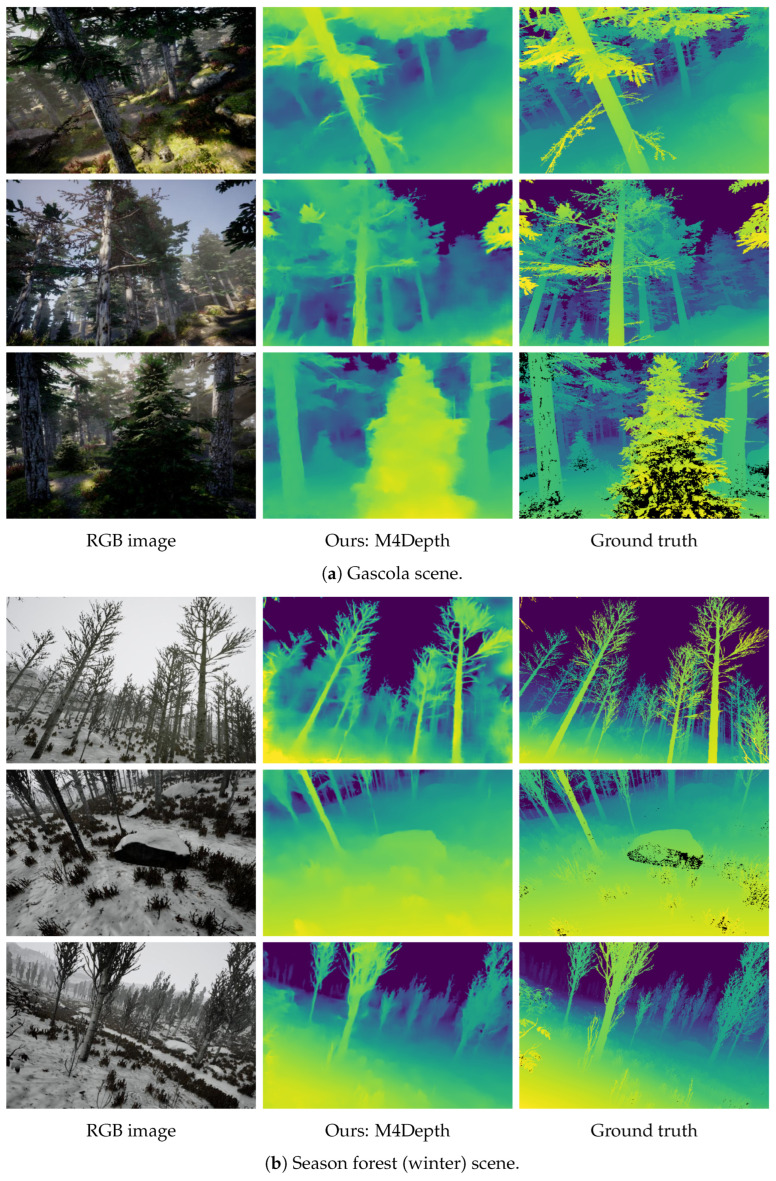
Samples of depth maps produced in generalization on unstructured scenes of the TartanAir dataset by M4Depth with six levels trained on Mid-Air. Black areas in the ground truths correspond to pixels with no color information in the RGB image.

**Figure 7:**
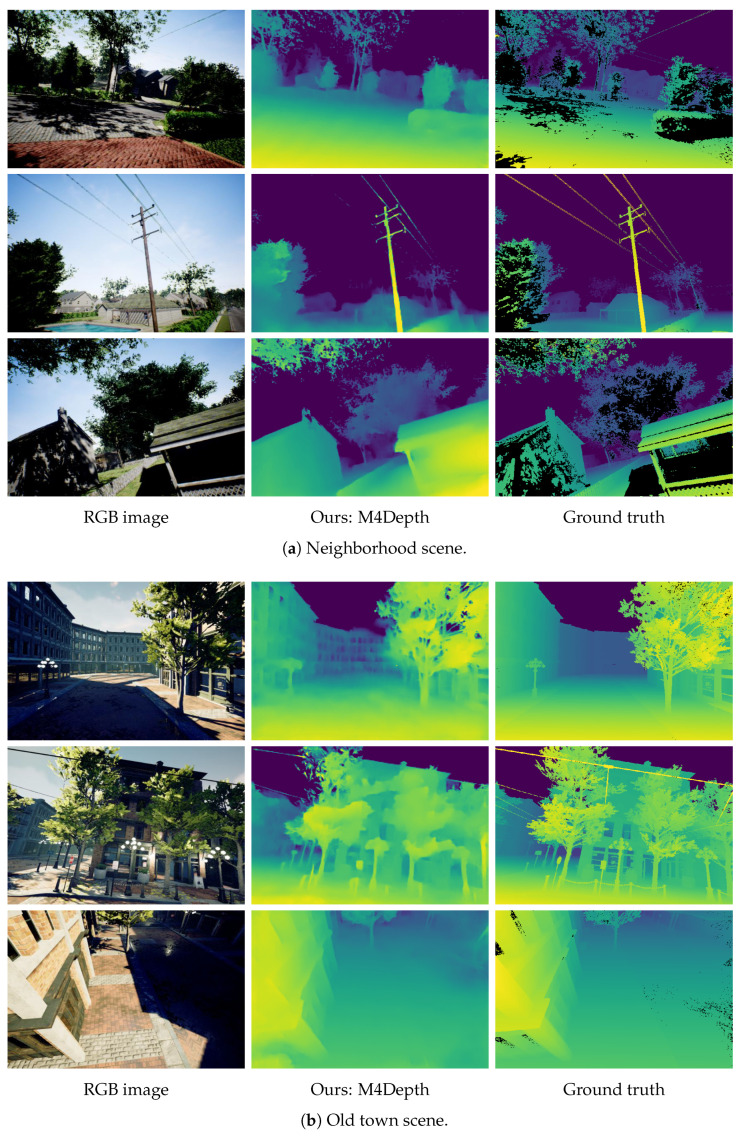
Samples of depth maps produced in generalization on urban scenes of the TartanAir dataset by M4Depth with six levels trained on Mid-Air and fine-tuned on KITTI. Black areas in the ground truths correspond to pixels with no color information in the RGB image.

The images obtained in generalization on TartanAir are shown in Figure 6 and Figure 7. It can be seen that the visual quality of the outputs in generalization is similar to that of the outputs produced on the dataset used for training. This confirms the strong performance of M4Depth in generalization as established with metrics.

Some general observations on the weaknesses of M4Depth can also be made from these outputs. First, the network cannot resolve all the details when the scene is too cluttered. This is especially visible in forest environments where tree branches overlap. Second, sometimes there are issues with sky recognition. This, however, is to be expected as our network mostly relies on perceived frame-to-frame pixel displacement to produce estimates. Finally, small and isolated structures such as cables are not always detected (see the outputs on urban scenes).

### 5.4. Discussion on the Architecture

**Ablation study.** We report the average performance over four trainings for ablated versions of our architecture in Table 6. The results show that the SNCV is the block that leads to the best performance boost. This highlights the benefits of giving some spatial information to the parallax refiners. The other blocks contribute to improve either test or generalization performances, but not both at the same time. As expected, the main contributors to generalization performances are the DINL and the normalization layer.

Increasing the number of levels in the architecture improves the performances. It should be noted, however, that the network tends to overfit the training dataset, therefore leading to worse generalization performance if the network gets too deep.

Overall, this ablation study shows that a compromise between performance on the training dataset and performance in generalization has to be made.

**Limitations.** With our approach, large areas with no repetitive textures are prone to poor depth estimates. The feature matching performed by our cost volumes matching can indeed become unstable if large areas share exactly the same features. This can therefore lead to bad depth estimates.

We mitigated this issue by using a multi-scale network and by including an SNCV at each of its levels, but these solutions do not make our network totally immune to this issue.

**Inference speed.** Our network has 4.5 million parameters and requires up to 500 MB of GPU memory to run with six levels. At inference time on Mid-Air, an NVidia Tesla V100 GPU needs 17 ms to process a single frame for a raw TensorFlow implementation. This corresponds to 59 frames per second which is roughly twenty-times faster than DeepV2D, the best-performing method on KITTI. According to NVidia’s technical overview [59], this should translate to 5 fps, at least, on their Jetson TX2 embedded GPU. Such inference speed is compatible with the real-time constraints required for robotic applications and can even be improved with inference optimizers such as TensorRT.

**Interpretation of the results.** As opposed to other methods, our network is designed to exclusively use the relative difference between feature vectors rather than relying on the raw semantic cues, i.e., the raw value of the feature vectors, to estimate depth. All reference methods, even the ones based on cost volumes, forward the feature maps generated by their encoder directly to their depth estimation subnetwork. Doing so gives networks the ability to use semantic cues directly to estimate depth. This ability is only valuable for instances where the set of features possibly encountered can be encoded by the network and associated to a specific depth.

Our experiments show that reference methods perform well—better than M4Depth for some—on KITTI, the dataset with constrained and structured scenes. However, they fall behind in unstructured environments when the link between semantic cues and depth is weak, and in generalization when semantic cues are different from the reference. This tends to imply that baseline networks rely on the raw feature values to derive depth.

All these observations lead us to believe that severing the direct link between the encoder and the decoder of the architecture while proposing relevant substitute data through the preprocessing unit is the key factor that allows M4Depth to perform so well overall in our experiments.

## 6. Conclusions

In this paper, we address the challenging task of estimating depth from RGB image sequences acquired in unknown environments by a camera moving with six degrees of freedom. For this, we first define a notion of visual parallax for generic camera motion, which is central for our M4Depth method, and we then show how it can be used to estimate depth. Then, we present new cost volumes designed to boost the performance of the underlying deep neural network of our method.

Three series of experiments were performed on synthetic datasets as well as on the KITTI dataset that features real data. They show that M4Depth is superior to the baseline both in unstructured environments and in generalization while also performing well on the standard KITTI benchmark, which shows its superiority for autonomous vehicles that would need to venture off road. In addition to being motion- and feature-invariant, our method is lightweight and fast enough to be considered for real-time applications.

Our further works on M4Depth will, among others, focus on the determination of its own uncertainty on depth estimates at inference time. Such an addition would provide a great advantage over other methods that do not offer this capability.

To help the community to reproduce our results, we made the code of our method publicly available at https://github.com/michael-fonder/M4Depth.

## Figures and Tables

**Table 1:** Main characteristics of a selection of depth estimation methods used for comparison in this paper.

Method	Supervision	Multi-Frame	Recurrent	Cam. Pose	Pre-Trained on KITTI
Monodepth [16]	Self-sup.	No	No	No	Available
Monodepth2 [17]	Self-sup.	No	No	No	Available
ST-CLSTM [27]	Self-sup.	No	Yes	No	Not available
Wang [25]	Self-sup.	No	Yes	No	Not available
ManyDepth [22]	Self-sup.	Yes	No	Self-est.	Available
DeepV2D [42]	Supervised	Yes	No	Self-est.	Available
Our method (M4Depth)	Supervised	Yes	Yes	Given	Not applicable

**Table 2:** Performance comparison on our test set of Mid-Air. Here, a 6-level version of M4Depth is compared to the baseline methods. Scores correspond to the best performance obtained out of five individual network trainings. The best score for a metric is highlighted in bold and the second best is underlined. The four first metrics need to be minimized (↓) and the three last ones need to be maximized (↑).

Method	Test Size	Abs Rel ↓	SQ Rel ↓	RMSE ↓	RMSE log ↓	δ<1.25 ↑	$\delta <1.25^{2}$ ↑	$\delta <1.25^{3}$ ↑
Monodepth [16]	384×384	0.314	8.713	13.595	0.438	0.678	0.828	0.895
Monodepth2 [17]	384×384	0.394	5.366	12.351	0.462	0.610	0.751	0.833
ST-CLSTM [27]	384×384	0.404	6.390	13.685	0.438	0.751	0.865	0.911
Wang [25]	384×384	0.241	5.532	12.599	0.362	0.648	0.831	0.911
ManyDepth [22]	384×384	0.203	3.549	10.919	0.327	0.723	0.876	0.933
PWCDC-Net [44]	384×384	**0.095**	**2.087**	8.351	0.215	0.887	0.938	0.962
M4Depth-d6 (Ours)	384×384	0.105	3.454	**7.043**	**0.186**	**0.919**	**0.953**	**0.969**

**Table 3:** Performance of M4Depth (best of 5 trainings) on the KITTI dataset. The scores reported for reference methods are the ones published by their respective authors. The best score for a metric is highlighted in bold and the second best is underlined. The four first metrics need to be minimized (↓) and the three last ones need to be maximized (↑).

Method	Test Size	Abs Rel ↓	SQ Rel ↓	RMSE ↓	RMSE log ↓	δ<1.25 ↑	$\delta <1.25^{2}$ ↑	$\delta <1.25^{3}$ ↑
Monodepth [16]	256×512	0.114	0.898	4.935	0.206	0.861	0.949	0.976
Monodepth2 [17]	320×1024	0.106	0.806	4.630	0.193	0.876	0.958	0.980
ST-CLSTM [27]	375×1240	0.104	N/A	4.139	0.131	0.833	0.967	0.988
Wang [25]	128×416	0.077	0.205	**1.698**	0.110	0.941	0.990	**0.998**
ManyDepth [22]	320×1024	0.087	0.685	4.142	0.167	0.920	0.968	0.983
DeepV2D [42]	300×1088	**0.037**	**0.174**	2.005	**0.074**	**0.977**	**0.993**	0.997
PWCDC-Net [44]	256×768	0.152	2.015	5.883	0.251	0.828	0.920	0.956
M4Depth-d6 (Ours)	256×768	0.095	0.7084	3.515	0.146	0.898	0.962	0.982

**Table 4:** Performance for the generalization test on two unstructured environments from TartanAir, that are gascola (G) and season forest winter (W). Scores were generated by using the same network weights as the ones used to report the performance on Mid-Air in Table 2. The best score for a metric is highlighted in bold and the second best is underlined. The four first metrics need to be minimized (↓) and the three last ones need to be maximized (↑).

Method	Test Size	Abs Rel ↓		SQ Rel ↓		RMSE ↓		RMSE log ↓		δ<1.25 ↑		$\delta <1.25^{2}$ ↑		$\delta <1.25^{3}$ ↑	
		G	W	G	W	G	W	G	W	G	W	G	W	G	W
Monodepth [16]	384×512	0.929	1.765	21.950	147.3026	19.116	33.162	0.992	1.118	0.231	0.224	0.430	0.384	0.590	0.516
Monodepth2 [17]	384×384	0.922	1.651	19.274	67.815	18.527	24.543	0.799	1.058	0.310	0.286	0.507	0.437	0.651	0.561
ST-CLSTM [27]	384×384	2.967	2.552	51.305	40.452	32.453	27.338	0.978	0.878	0.375	0.370	0.517	0.518	0.626	0.609
Wang [25]	384×512	0.761	0.776	25.459	29.138	31.875	28.332	1.482	1.260	0.209	0.259	0.313	0.353	0.411	0.442
ManyDepth [22]	384×384	0.776	1.383	16.551	63.285	16.822	23.607	0.746	0.974	0.326	0.374	0.538	0.544	0.684	0.654
PWCDC-Net [44]	384×512	0.343	**0.516**	7.645	18.459	17.731	30.028	0.684	1.160	0.584	0.463	0.716	0.568	0.786	0.632
M4Depth-d6 (Ours)	384×512	**0.281**	0.537	**5.348**	**17.040**	**11.875**	**16.937**	**0.524**	**0.694**	**0.715**	**0.663**	**0.806**	**0.746**	**0.856**	**0.798**

**Table 5:** Performance for the generalization test on two structured environments from TartanAir, that are neighborhood (N) and old town (OT). Scores were generated by using the same network weights as the ones used to report the performance on KITTI in Table 3. The best score for a metric is highlighted in bold and the second best is underlined. The four first metrics need to be minimized (↓) and the three last ones need to be maximized (↑).

Method	Test Size	Abs Rel ↓		SQ Rel ↓		RMSE ↓		blue!25RMSE log ↓		δ<1.25 ↑		$\delta <1.25^{2}$ ↑		$\delta <1.25^{3}$ ↑	
		N	OT	N	OT	N	OT	N	OT	N	OT	N	OT	N	OT
Monodepth [16]	384×512	1.041	0.909	54.683	30.616	30.957	19.203	0.843	0.782	0.261	0.267	0.465	0.489	0.620	0.655
Monodepth2 [17]	320×1024	0.810	0.775	27.904	17.973	21.011	15.800	0.732	0.727	0.412	0.322	0.603	0.540	0.715	0.692
ManyDepth [22]	320×1024	0.942	0.759	42.846	20.895	22.508	15.604	0.757	0.649	0.432	0.351	0.607	0.583	0.714	0.730
DeepV2D [42]	384×512	1.5157	0.694	77.063	18.777	21.546	7.551	0.769	0.498	0.335	0.494	0.527	0.722	0.641	0.830
PWCDC-Net [44]	384×512	**0.376**	0.338	**12.66**	8.679	23.782	16.760	0.788	0.703	0.535	0.627	0.652	0.741	0.723	0.800
M4Depth (Ours)	384×512	0.509	**0.256**	24.283	**6.759**	**13.150**	**7.211**	**0.502**	**0.370**	**0.749**	**0.804**	**0.827**	**0.880**	**0.872**	**0.918**

**Table 6:** Performance of M4Depth (trained on Mid-Air, averaged over 4 runs) for various architecture depths and ablations (on a network with 6 levels), and for a full architecture with 2, 4, and 6 levels, when tested on Mid-Air (MA) as well as in generalization on the old town scene (OT) of TartanAir. The four first metrics need to be minimized (↓) and the three last ones need to be maximized (↑).

Ablation	Abs Rel ↓		SQ Rel ↓		RMSE ↓		RMSE log ↓		δ<1.25 ↑		$\delta <1.25^{2}$ ↑		$\delta <1.25^{3}$ ↑	
	MA	OT	MA	OT	MA	OT	MA	OT	MA	OT	MA	OT	MA	OT
SNCV	0.118	0.609	4.392	26.870	7.730	9.469	0.203	0.608	0.912	0.738	0.947	0.807	0.965	0.846
Normalize	0.099	0.583	3.179	23.495	7.032	9.019	0.185	0.496	0.920	0.770	0.955	0.842	0.971	0.880
DINL	0.104	0.521	3.480	19.378	7.182	8.826	0.189	0.536	0.915	0.763	0.952	0.833	0.969	0.872
$f_{\rho ,t}^{l-1}$	0.113	0.435	4.007	14.445	7.382	8.732	0.196	0.517	0.916	0.771	0.950	0.840	0.967	0.878
Split	0.113	0.366	4.074	9.937	7.424	7.900	0.196	0.478	0.914	0.762	0.949	0.834	0.966	0.873
$\rho_{t-1}^{l}$	0.107	0.435	3.482	15.071	7.201	8.836	0.197	0.437	0.911	0.788	0.949	0.863	0.966	0.900
M4Depth-d2	0.108	0.660	3.164	30.091	8.141	13.743	0.230	0.618	0.903	0.742	0.943	0.820	0.962	0.861
M4Depth-d4	0.114	0.330	4.124	12.569	7.405	8.391	0.196	0.399	0.916	0.809	0.951	0.882	0.967	0.917
M4Depth-d6	0.109	0.434	3.724	14.087	7.169	8.875	0.190	0.494	0.917	0.778	0.952	0.848	0.968	0.885

**Figure A1:**
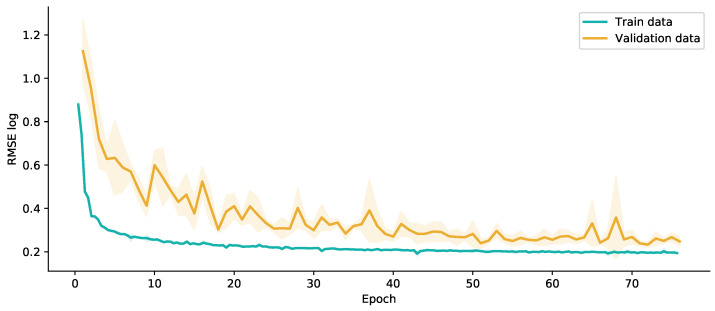
Overview of the performance of M4Depth during its training on the Mid-Air dataset. The reported performances are the ones of the network on the train and validation sets. The lines and the colored areas indicate the average performance of the network over four training and its standard deviation, respectively.

## Data Availability

All the datasets used in this work are public. The Mid-Air, Kitti, and TartanAir datasets can be respectively found at the following URLs: https://midair.ulg.ac.be/, https://www.cvlibs.net/datasets/kitti/, and https://theairlab.org/tartanair-dataset/. The code used to generate the results presented in this paper is publicly available on GitHub at https://github.com/michael-fonder/M4Depth. All the URLs mentioned here were accessed on 3 November 2022.

## Appendix A. Mid-Air Training Details (Complement to Section 5.1 Unstructured Environments)

In this section, we provide an additional overview on the performance of M4Depth during its training on the Mid-Air dataset as well as the training details required to reproduce the results of the methods used for our baseline on the Mid-Air dataset. These details complement the code made available on our GitHub repository.

We report the performance of M4Depth during its training on the Mid-Air dataset in Figure A1. As expected, the average error on the training set decreases monotonously over time. The error on the validation set also tends to decrease, while being noisier. The important factor to notice is that the performance in validation remains stable at the end of the training, which indicates that the network does not overfit the training set.

In the paper, we have chosen six methods for our baseline, namely: Monodepth [16,60], Monodepth2 [17,61], ST-CLSTM [27,62], the method of Wang et al. [25], Wang et al. [63], ManyDepth [22,64], and PWCDC-Net [44].

To get a baseline that is true to the work of the authors and is coherent between different methods, we proceeded as follows. We retained the original default parameter values of each method. Next, we adjusted the batch size so that every learning step contained around 18 frames, and we trained each network five times. As some methods have specific input pipelines, we adjusted the training epoch count of each method to guarantee that a network sees every training sample at least 50 times during its training. After a first round of training, it appeared that some methods did not converge. This led us to adapt their training setup to get a representative performance.

The training of PWCDC-Net [44] had to be carried out differently as it is an optical flow network. To make it work, we had to convert depth maps to optical flow maps by using Equation (7). It also required more steps during the training to reach a steady-state on the validation set.

**Table A1:** Main parameters used to train baseline methods. The asterisk denotes default parameters suggested by authors for their respective method.

Method	Train Epoch Count	Batch Size	Sequence Length
Monodepth [16,60]	50	18	1 *
Monodepth2 [17,61]	17	6	3 *
ST-CLSTM [27,62]	50	3	5 *
Wang et al. [25,63]	50	3	8
ManyDepth [22,64]	25	6	3 *
PWCDC-Net [44]	100	8	2 *

The values reported for the baseline methods correspond to the best results obtained out of five runs. The most important parameters of the baseline setup are given in Table A1. All other parameters remained unchanged to a large extent. However, adjustments were necessary for some methods as explained hereafter.

- With the proposed setup, Monodepth failed to produce any output for three trainings out of the five.
- The lower epoch count for Monodepth2 is due to the fact that the training pipeline sees each sample three times during a single epoch.
- Performances obtained with the default learning rate for the ST-CLSTM method were extremely poor. We obtained better results by reducing it to $10^{-4}$.
- The code written by Wang et al. [63] worked as expected. However, the length of the training sequence had to be downsized from 10 to 8 frames to accommodate our internal pipeline constraints.
- Finally, for Manydepth, we had to select the encoder architecture; we chose the ResNet-50 encoder.
